# A facile approach to synthesize SiO_2_ · Re_2_O_3_ (Re = Y, Eu, La, Sm, Tb, Pr) hollow sphere and its application in drug release

**DOI:** 10.1186/1556-276X-8-435

**Published:** 2013-10-21

**Authors:** Zhihua Li, Lin Zhu, Qian Liu, Yu Du, Feng Wang

**Affiliations:** 1 Department of Chemistry, College of Chemistry, Chemical Engineering and Materials Science, Shandong Normal University, Jinan 250014, China; 2 College of Rizhao Polytechnic, Rizhao 276826, China

**Keywords:** SiO_2_ · Re_2_O_3_, Hollow spheres (HSs), Fluorescence

## Abstract

Multifunctional SiO_2_ · Re_2_O_3_ (Re = Y, Eu, La, Sm, Tb, Pr) hollow spheres (HSs) have been fabricated using an acidic Re^3+^ ion solution. Under ultraviolet radiation, functional HSs emit different colors of light according to the different rare-earth ions embedded into the shell of SiO_2_ hollow spheres. The as-prepared hollow capsules were characterized by X-ray diffraction, transmission electron microscopy, high-resolution transmission electron microscopy, Brunauer-Emmett-Teller method, scanning electron microscopy, and energy-dispersive spectrometry. Drug loading and release experiments have been carried out using SiO_2_ · Eu_2_O_3_ HSs that acted as drug carriers. The results demonstrate that the multifunctional HSs exhibit a high storage capacity and the ability of retaining drug stability and activity, which indicates that the as-synthesized fluorescent hollow capsules are a potential candidate as drug delivery materials.

## Background

Recently, much attention have been focused on the research of hollow SiO_2_ spheres (HSSs) because of their excellent properties such as thermal stability, large surface areas, low density, low toxicity, and good compatibilities with other materials [1-15]. So, HSSs have attracted intense interest and have been widely applied in a variety of fields, such as catalysis, sensors, chromatography, dyes, inks, photonic crystals, cells, waste removal, shield for enzymes or proteins, delivery vehicle of drugs, and large biomolecular release [16-28]. In general, three approaches are employed to prepare HSSs: template methods [6,29-31], self-assembly technique, and microemulsions [32,33]. HSSs [34-36] have been fabricated with the soft template and hard template methods, which involve complicated procedures such as shell formation and core removal. The template-free method has attracted much attention due to its simple and economical characteristic [27,28]. In 2008, Zhang et al. [25] developed a self-template method to convert solid silica into hollow spheres (HSs). In the process, silica is dissolved into NaBH_4_ solution and silicate species deposited on the surface of the silica colloid. The shell formed over the silicate species via Ostwald ripening results in HSSs. An alkalescent environment is an inevitable synthesis condition that is reported in nearly all papers [37-50]; the only exception is that of Chen et al. who used HF as an etching agent [51]. In 2011, Wang’s group reported firstly the synthesis of HSSs in generic acidic media [52]. Unfortunately, the published work was based on the study of only two factors: pH value and Na^+^ ion, which influence the formation process of HSSs.

Herein, we report a one-step self-driven process to synthesize multifunctional HSSs under an acidic condition with rare-earth ion assistance. Compared with Wang’s report, the synthetic approach of HSSs is simpler. Being synthesized with the assistance of rare-earth ions, the as-prepared HSSs can emit bright fluorescence under ultraviolet radiation, which is convenient to be detected in real time if it is used in biological applications. Typical drug loading and release experiments are carried out using our prepared multifunctional HSSs, SiO_2_ · Eu_2_O_3_ HSs.

## Methods

All chemicals were of analytical grade and purchased from Jinan Camolai Trading Company (Jinan, China), which were used as received without further purification: tetraethyl orthosilicate (TEOS, 98%), ammonium hydroxide solution (NH_3_ · H_2_O, approximately 25% in water), nitric acid (HNO_3_, 65%), Re_2_O_3_, and ethanol (C_2_H_5_OH). Rare-earth nitrate solutions [Re(NO_3_)_3_ (Re = Y, Eu, La, Sm, Tb, Pr)] with a concentration of 0.04 to 0.08 mol/L were prepared by ourselves.

### Synthesis of monodisperse silica spheres

Silica spheres with a diameter of 200 to 500 nm were prepared by the hydrolysis of TEOS in the mixture of ethanol, water, and ammonium using the Stöber process [37-39].

### Synthesis of SiO_2_ · Re_2_O_3_ hollow spheres

In a typical synthesis, silica spheres (0.06 g) were added to 20 mL Re(NO_3_)_3_ (0.06 mol/L) and stirred for 30 min. The pH of the solution is 4.5 (adjusted with dilute nitric acid). The mixture was transferred into a Teflon-lined stainless autoclave (capacity 25 mL) and heated at 250°C for 12 h. After the products naturally cooled down to room temperature, they were washed with deionized water and separated by centrifugation (4,000 rpm) for three times and then dried at 60°C for 4 h in the air.

### Drug storage and release

The steps of drug storage and release are as follows:

1. SiO_2_ · Eu_2_O_3_ HSs (1 g) were added into a 50-mL hexane solution containing 40 mg/mL ibuprofen (IBU). The mixture was sealed and stirred for 24 h. Then the sample was separated by centrifugation and dried at 60°C in the air. The filter was characterized by UV-visible (UV–vis; 264 nm) spectroscopy.

2. The dry SiO_2_ · Eu_2_O_3_ loaded with IBU (0.1 g) was immersed into 50 mL of simulated body fluid (SBF; pH = 7.4) at 37°C and stirred at the rate of 100 rpm. Three milliliters from the top of the solution was used for release measurement at different intervals, and then 3 mL of fresh SBF is added into the solution to keep the volume unchanged.

### Characterization and instruments

The characterization and instruments used are detailed as follows:

1. The samples were characterized by X-ray diffraction (XRD) with a Philips X’Pert Super diffractometer (Amsterdam, The Netherlands) with graphite-monochromatized Cu Kα radiation (*λ* = 1.54178 Å) in the 2*θ* range of 1.5° to 10° and 10° to 80°.

2. The transmission electron microscopy (TEM) images and electron diffraction (ED) patterns were obtained using a Hitachi 800 transmission electron microscope (Chiyoda-ku, Japan) with an accelerating voltage of 200 kV.

3. The high-resolution transmission electron microscopy (HRTEM) images were obtained using JEOL-2010 (Akishima-shi, Japan).

4. The UV–vis absorption spectra of the samples were measured using a UV-1800 ultraviolet–visible spectrophotometer (Shanghai Meipuda Instrument Co., Ltd., Shanghai, China).

The samples used for characterization were ultrasonically dispersed in absolute ethanol for 30 min before the TEM and HRTEM tests.

## Results and discussion

### Characterization of SiO_2_ · Eu_2_O_3_ HSs

**Figure 1:**
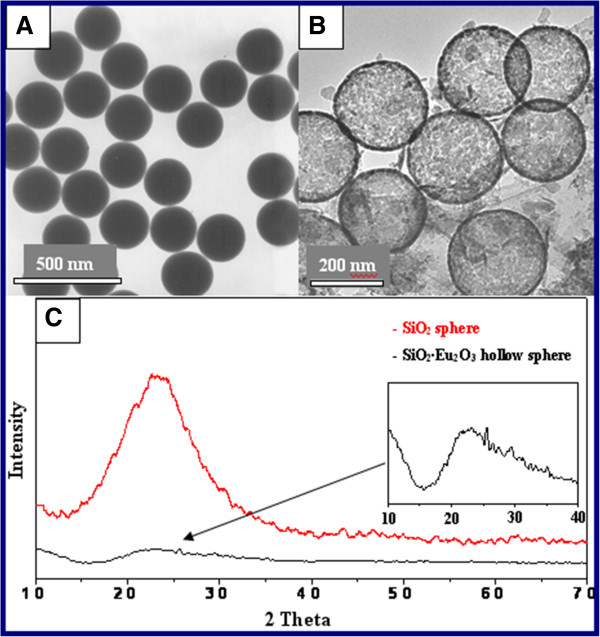
**TEM image of SiO**_**2 **_**sphere (A), HRTEM image of SiO**_**2**_**∙Eu**_**2**_**O**_**3 **_**HSs (B), XRD patterns of SiO**_**2 **_**sphere and SiO**_**2**_**∙Eu**_**2**_**O**_**3 **_**HSs (C).** The insert is magnification of one segment of XRD.

**Figure 2:**
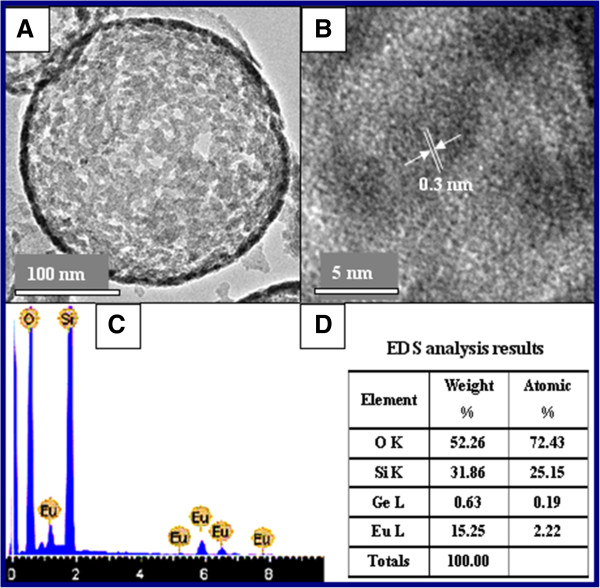
**HRTEM images and EDS pattern of SiO**_**2**_ **· Eu**_**2**_**O**_**3 **_**HSs. (A)** Mesoporous structure of SiO_2_ · Eu_2_O_3_. **(B)** The interplanar spacing of the (001) plane of Eu_2_O_3_. **(C, D)** EDS pattern and results of SiO_2_ · Eu_2_O_3_ HSs, respectively.

Newly prepared silica spheres were used to fabricate HSSs. The monodispersed SiO_2_ spheres with an average diameter of 230 nm (Figure 1A) were fabricated using the Stöber method [37-39] and acted as the template. The hollow SiO_2_ · Eu_2_O_3_ HSs were uniform, as shown in the HRTEM image in Figure 1B, whose size was nearly unchanged. XRD curves in Figure 1C demonstrate that both the SiO_2_ sphere and SiO_2_ · Eu_2_O_3_ hollow sphere are amorphous (compared with ICSD #174). The absence of diffraction peaks for Eu_2_O_3_ was owing to the few content of Eu_2_O_3_ in the sample. Figure 2 shows the HRTEM image and energy-dispersive spectrometer (EDS) analysis of SiO_2_ · Eu_2_O_3_ HSs. A large number of holes with different sizes on the surface of SiO_2_ · Eu_2_O_3_ HSs could be observed in Figure 2A, which belonged to a range of mesoporous structures according to the diameter of holes. The SiO_2_ · Eu_2_O_3_ HSs with numerous mesoporous structures indicated that they are potential drug carriers for application in medicine, e.g., targeting therapy. The results of the EDS analysis showed that the content of O, Si, and Eu was 72.43%, 25.15%, and 2.22%, respectively. The microcontent of Ge (0.19%) was due to the impurity coming from the reagent of Eu_2_O_3_. The SiO_2_ HSs were amorphous according to their XRD pattern, so the lattice fringe that appeared on the HRTEM image (Figure 2B) stemmed from Eu_2_O_3_. The measured interplanar spacing of 0.3 nm corresponded to the (001) plane of Eu_2_O_3_. Obviously, Eu_2_O_3_ is one component of the final product, and it may be embedded into the shells or form a kind of composite similar to ‘alloy’ or a solid solution. Further research is in progress. Being doped with Eu_2_O_3_ on the surface of SiO_2_ HSs, the obtained samples can emit bright red light under an ultraviolet beam. HRTEM observation also revealed that the HSs produced in the solution contained Re^3+^ ions that formed a mesoporous structure with different orientations.

**Figure 3:**
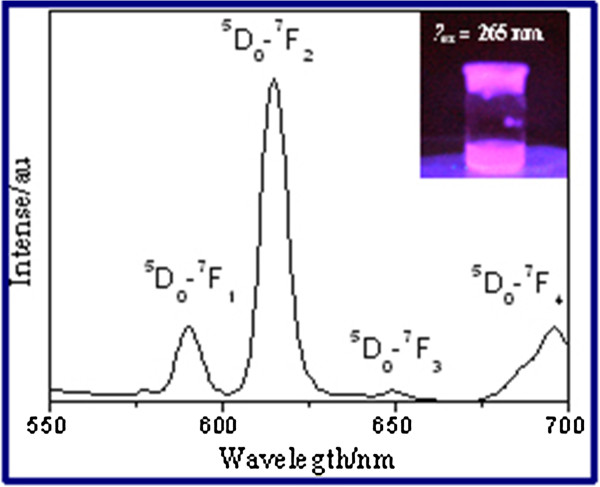
**The emission spectrum of SiO**_**2**_**∙Eu**_**2**_**O**_**3 **_**HSs.** The insert is digital image of SiO_2_∙Eu_2_O_3_ HSs under UV light.

The measured emission spectrum curve of SiO_2_ · Eu_2_O_3_ HSs is shown in Figure 3. The strong red emission peak further suggested that Eu^3+^ existed in the surface of the SiO_2_ hollow sphere. The emission spectrum of SiO_2_ · Eu_2_O_3_ HSs consisted of peaks mainly located in the wavelength range from 570 to 700 nm. These peaks corresponded to transitions from the excited state ^5^*D*_0_ to the ground state ^7^*F*_*J*_ (*J* = 0, 1, 2, 3, 4) of the 4*f*^6^ configuration of Eu^3+^, as marked in Figure 3. Luminescence originating from transitions between 4*f* levels is predominant due to electric dipole or magnetic dipole interactions [40-44]. As can be seen in Figure 3, the strong red emission peak at 612 nm originating from the electric dipole transition ^5^*D*_0_ to ^7^*F*_2_ was the dominant band in the measured spectrum. The emission peak at around 590 nm was attributed to the ^5^*D*_0_ to ^7^*F*_1_ transition. The peaks located at 648 and 695 nm corresponded to ^5^*D*_0_ to ^7^*F*_3_ and ^5^*D*_0_ to ^7^*F*_4_ transitions, respectively.

**Figure 4:**
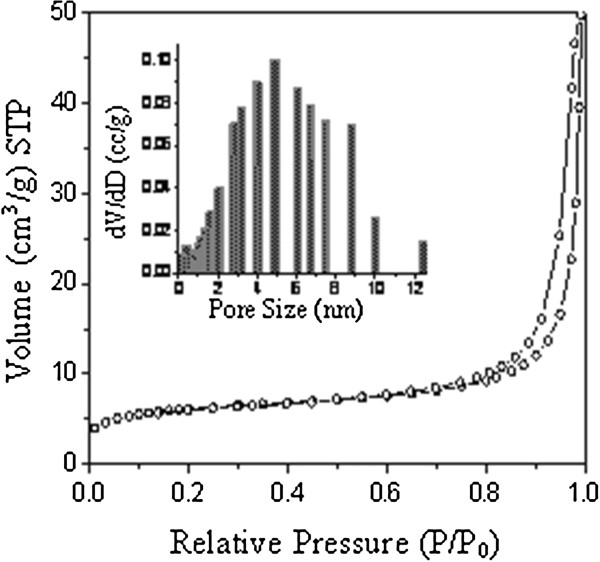
**N**_**2 **_**adsorption-desorption isotherm and pore size distribution (insert) of SiO**_**2**_**∙Eu**_**2**_**O**_**3 **_**HSs.**

Figure 4 shows the Brunauer-Emmett-Teller (BET) N_2_ adsorption-desorption isotherms and the pore size distribution of the as-prepared SiO_2_ · Eu_2_O_3_ HSs. The BET specific surface area and the total pore volume of the SiO_2_ · Eu_2_O_3_ HSs were measured to be 308.6 m^2^/g and 0.307 cm^3^/g, respectively. The pore diameter distribution was relatively wide according to the data of the adsorption branch of the isotherm. The as-prepared SiO_2_ · Eu_2_O_3_ HSs with a mesoporous structure may possess good performance in drug delivery efficiency, catalytic activity, and so on.

### Influencing factors of the synthetic process of SiO_2_ · Re_2_O_3_ (Re = Y, Eu, La, Sm, Tb, Pr) hollow structures

The experiments showed that the pH value of the solution, reaction temperature and time, and different rare-earth ions and concentrations played an outstanding role in the synthesis of SiO_2_ · Re_2_O_3_ hollow structures, which are discussed in detail as follows.

#### Effect of the pH value of the solution

The pH value of the solution was adjusted with dilute nitric acid. The studied pH range was from 7 to 3 under the following reaction conditions: Re^3+^ = 0.06 mol/L and *T* = 250°C. Hollow-structure particles could be obtained under the range of 4 ≤ pH < 5.5, and the optimum pH value was 4.5. No hollow structure products appeared when 6 ≤ pH ≤ 8. No HSSs appeared when 2 < pH < 3. Normally, a few HSSs could have emerged in the product at the conditions of 3 < pH < 4.0 or 5 < pH < 6 if the reaction time was more than 10 h. The detailed results are shown in Additional file 1: Table S1 and Figure S3. It is known that SiO_2_ is an amphoteric oxide which can dissolve into an acidic or basic solvent. The experiments showed that a weak acid solution was in favor of hollow structure formation. The experiments also revealed that the experimental results were unaffected when using other acids such as HCl or H_2_SO_4_ to adjust the pH value of the solution.

#### Effect of reaction temperature

The temperature of the hydrothermal reaction affected greatly not only the reaction (going or not) but also the reaction rate (slow or fast). Additional file 1: Figure S1 shows the TEM images of the as-prepared samples at different reaction temperatures. No hollow-structure products appeared if the temperature *T* < 230°C in our experiments. The morphology and size of nanocrystals became difficult to control when the temperature was up to 260°C or higher because the higher the temperature was, the faster the reaction rate was. When *T* = 255°C, the quality of the obtained SiO_2_ · Re_2_O_3_ HSs was always poor. The experiments verify that the moderate temperature was 250°C.

#### Effect of Re^3+^ ion and its concentration

It was reported that Na_2_SO_4_ and NaCl were advantageous to HSS formation [52] and the work matter was Na^+^ cation, which was in line with our experimental data. Hereby, we investigated the synthesis of HSSs under different rare-earth ions and bivalent cations.

In order to get uniform hollow structures, the optimal concentration of the rare-earth ions was usually kept in the range of 0.04 to 0.08 mol/L. The experimental data and TEM images are depicted in Additional file 1: Table S1 and Figure S2. The concentration less than 0.03 mol/L resulted in poor quality in production, and the concentration greater than 0.08 mol/L always led to products with not all having a hollow structure. The experiments showed that the lower or higher concentration of Re^3+^ ions was not good for HSS formation and 0.06 mol/L was the optimal concentration.

Although the SiO_2_ · Re_2_O_3_ HSs were obtained based on the rare-earth ion assistance strategy, their quality was quite different under assistance of different kinds of rare-earth ions. By keeping other reaction conditions unchanged such as the pH value of the solution, reaction time, and reaction temperature, the influence of different Re^3+^ ions (Re = Y, Eu, La, Sm, Tb, Pr) on the structure of the as-prepared products was investigated (see Additional file 1: Table S2 and Figure S4). Additional file 1: Figure S4 clearly shows that the influence sequence of Re^3+^ was as follows: Eu^3 +^ ≈ Sm^3 +^ > Y^3 +^ > Pr^3 +^ ≈ La^3 +^ > Tb^3 +^. Nearly all of the as-prepared samples were hollow spheres with good quality under the effect of Eu^3+^ and Sm^3+^ existence, and the experiments showed good reproducibility and satisfactory results. With Y^3+^, Pr^3+^, and La^3+^ ions included, all of the products always formed a mixture of HSSs and core/shell structure. Furthermore, all of the samples can be formed into a hollow sphere if the reaction time is prolonged, but the yield of HSSs was lower. Only a small amount of HSs could be obtained with Tb^3+^ existence. The experiments indicated that changing the reaction time did not work.

We compared the synthesis approach under the same reaction conditions using Mg^2+^, Ca^2+^, Mn^2+^, Zn^2+^, and Cu^2+^ instead of Re^3+^ ions. The results showed that SiO_2_ · HSs could barely be obtained at the above situations, which indicated that rare-earth ion was an indispensable factor in hollow structure formation.

Experimental data showed that the rare-earth ions were advantageous to HSS formation; however, further study is needed to understand why the effect of different Re^3+^ ions on the formation of HSSs has a different role.

#### Effect of reaction time

**Figure 5:**
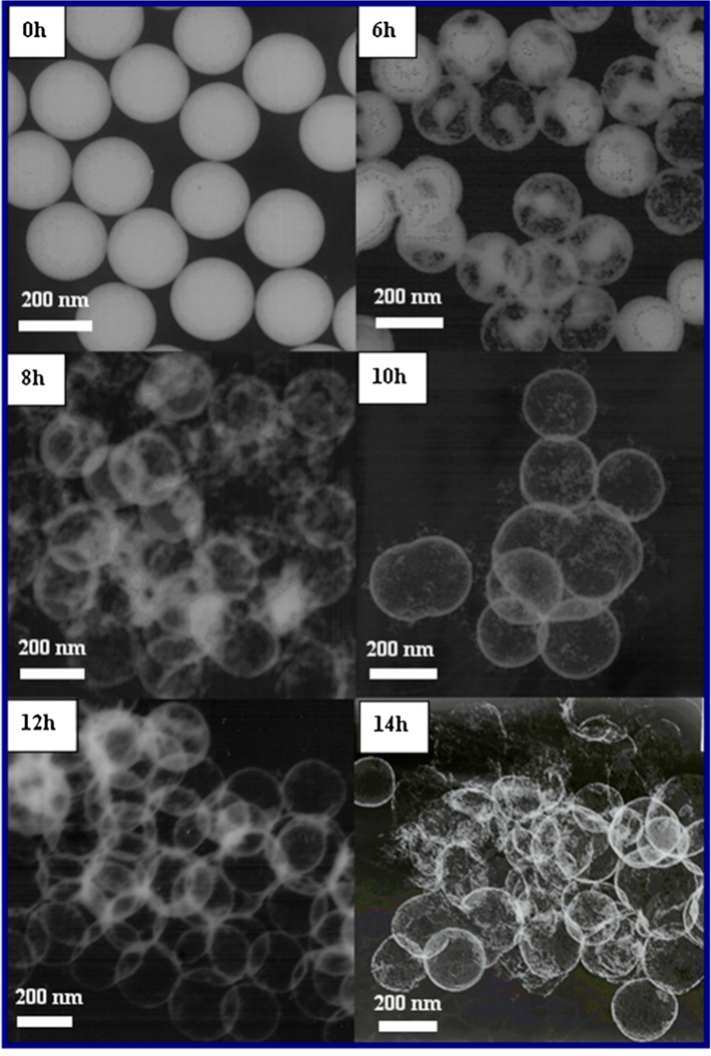
**TEM images of products prepared at different reaction time.** T = 250°C, pH = 4,[Eu^3+^] =0.06 mol/L.

The reaction time will determine the deepening of the reaction after fixing other reaction conditions. Figure 5 shows the structures of the as-prepared particles with a variety of reaction time. As can be seen, rattle-type particles appeared after 6 h of reaction, and then the core of particles gradually disappeared and finally became HSs at about 8 h, meanwhile many tiny particles accompanied with the HSs. After 10 h, the shapes of HSs were clearer, though many tiny particles were around them. The tiny particles came from the dissolved SiO_2_, which disappeared with reaction time extension. The high-quality HSs with clear edges were obtained when the reaction lasted for 12 h; simultaneously, the tiny particles disappeared too. It was noticed that the shell of hollow spheres was getting thinner and thinner when the reaction time was over 12 h. As can be seen, a handful of HSs had cracked after 14 h. The experiments indicated that the reaction time would significantly vary the influence on the shell thickness of SiO_2_ · Re_2_O_3_ HSs. Therefore, the shell thickness of HSs can be controlled by adjusting the reaction time.

From the above, our synthesis procedure of HSSs is very simple and effective compared with those previously reported.

### Formation mechanism of SiO_2_ · Re_2_O_3_ HSs

In our experiments, SiO_2_ · Re_2_O_3_ HSs were synthesized in an acidic solution. It was reported that colloid SiO_2_ would carry a negative charge when pH > 3 [45]. The following equilibriums existed in the intermediate between the liquid and solid interfaces [48]:

$${\mathrm{SiO}}_{2}\overset{H^{+}}{↔}{\mathrm{HSiO}}_{4}{}^{3-}\overset{H^{+}}{↔}H_{2}{\mathrm{SiO}}_{4}{}^{2-}\overset{H^{+}}{↔}H_{3}{\mathrm{SiO}}_{4}{}^{-}\overset{H^{+}}{↔}H_{4}{\mathrm{SiO}}_{4}\overset{H^{+}}{↔}H_{5}{\mathrm{SiO}}_{4}{}^{+}\cdot \cdot \cdot .$$

**Figure 6:**
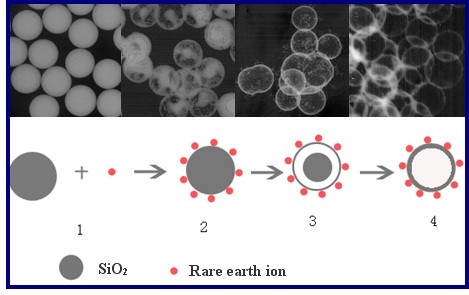
**Schematic diagram of the formation of SiO**_**2**_**∙Re**_**2**_**O**_**3 **_**HSs.**

When Re^3+^ ions are added into the solution, an electrostatic force is produced between the surface of silica and Re^3+^, Re^3+^ ions are absorbed onto the surface of SiO_2_ spheres at first, and then insoluble compounds SiO_2_ · Re_2_O_3_ are formed. Meanwhile, SiO_2_ cores keep dissolving in the acidic solution, as shown in Figure 5 (6 h). At the initial stage, most of the Re^3+^ ions are absorbed onto the surface of SiO_2_ spheres, and numerous insoluble tiny particles that come from the residual Re^3+^ ions meet with the negative ions in the solution, as shown in Figure 5 (8 and 10 h). As the reaction continues, the tiny particles are swallowed by the SiO_2_ · Re_2_O_3_ lamella due to Ostwald ripening, and clear SiO_2_ · Re_2_O_3_ HSs are obtained at last, as shown in Figure 5 (12 h). Figure 6 is the sketch map of SiO_2_ · Re_2_O_3_ HS formation. In the first step, Re^3+^ would be adsorbed on the surface of the silica colloid, and then the silica colloid turns into a rattle-type particle and finally turns into a HS through Ostwald ripening after being treated in hydrothermal conditions.

The experiments showed that the diameter of SiO_2_ · Re_2_O_3_ HSs was almost the same as that of the template, which indicated that the size of SiO_2_ · Re_2_O_3_ HSs was determined by the SiO_2_ solid spheres. Therefore, we can control the size of SiO_2_ · Re_2_O_3_ HSs by controlling the diameter of SiO_2_ solid spheres.

### Drug delivery and release

Considering that HSs have numerous mesoporous structures on the surface, they can act as drug loading capsules. IBU, a typical anti-inflammatory drug, is a good example used for drug loading experiments [49,53]. Herein, IBU was used to study the drug delivery and release behavior of nanostructured HSs.

The SiO_2_ · Re_2_O_3_ HSs were 1 g after loading IBU (see the ‘Methods’ section), and the IBU storage in nanostructured SiO_2_ · Re_2_O_3_ HSs reached 287.8 mg/g, which means that the as-prepared SiO_2_ · Re_2_O_3_ HSs have a high loading capacity.

**Figure 7:**
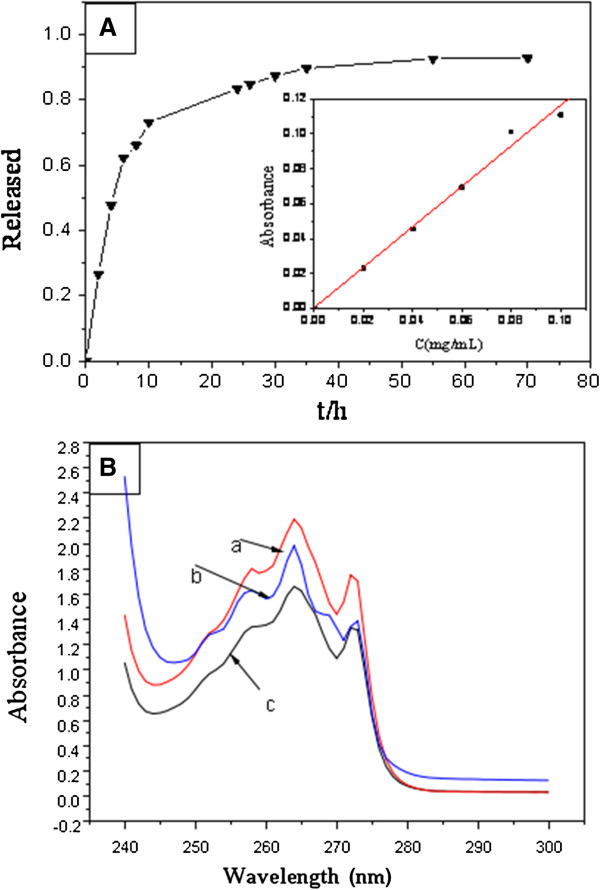
**Release efficiency and UV–vis absorption spectra of IBU. (A)** Release efficiency of IBU in the PBS system. The insert is the standard curve of CIBU absorbance. **(B)** The UV–vis absorption spectra of IBU in the different release times. Curve a, IBU hexane solution before drug loading; curve b, the SBF solution after the release of IBU-loaded SiO_2_ · Eu2O_3_ HSs for 4 h; curve c, the SBF solution after the release of IBU-loaded SiO_2_ · Eu_2_O_3_ HSs for 70.

The rate of drug release determines the drug effect. Slow and sustained drug release ensures a long drug effect. First of all, a phosphate buffer solution (PBS) of IBU (0.1 μg/mL) was prepared to find out the maximum absorption wavelength using a UV-visible spectrophotometer. The experiments indicated that the maximum absorption wavelength of IBU was 264 nm. According to the Lambert-Beer law, *A* = *kC*, where *A* is the absorbency, *k* is a constant, and *C* is the concentration of IBU in PBS. The insert of Figure 7A is the working curve of IBU in PBS, which was obtained by the measured absorbency of different PBS concentrations. The relationship between the concentration of IBU in PBS and absorbency was as follows:

$$\begin{array}{l} A=1.14951C\\ \;+0.00109\;\left(\mathrm{mg}/\mathrm{ml}\right),\mathrm{its}\;\mathrm{related}\;\mathrm{coefficient}R^{2}\\ \;=0.9948. \end{array}$$

The released IBU concentration in SBF could be calculated using the following equation:

$$C=\left(A-0.00109\right)/1.14915.$$

The total release rate of IBU can be calculated by the following equation:

$$R\left(\%\right)=\frac{C_{i}*50+\sum_{1}^{i-1}C_{i}*3}{m}$$

where *R* is the total release rate, *C*_*i*_ is the IBU concentration in SBF at time *i*, *i* is the time of the IBU medium solution taking out from the SBF, and *m* represents the total mass of the IBU in the HSs.

Figure 7A shows the release behavior of the IBU-loaded SiO_2_ · Eu_2_O_3_ HSs in SBF. Compared with the pure IBU disk release in SBF, the release rate of the IBU-loaded SiO_2_ · Eu_2_O_3_ HSs lasted long. The drug release rate was very fast within 12 h, which showed a nearly linear relationship between drug release rate and release time at the first 12 h. Then the drug release process was very slow and finished after 60 h. The results indicated that the sustained release behavior of the drug carrier was in favor of a durative drug effect.

In order to investigate the properties of the loaded drug, the UV–vis absorption spectra of the IBU hexane solution before and after IBU loading in SiO_2_ · Eu_2_O_3_ HSs were measured. The results are shown in Figure 7B. Curves a, b, and c were the IBU hexane solution before drug loading, the SBF solution after the release of IBU-loaded SiO_2_ · Eu_2_O_3_ HSs for 4 h, and the SBF solution after the release of IBU-loaded SiO_2_ · Eu_2_O_3_ HSs for 70 h, respectively. It was noticed that the shape of the absorption curves was essentially the same, which demonstrated that the property of IBU was not changed in the loading and release processes.

We noticed that the samples still emitted fluorescence after the experiments of drug delivery and release, which indicated that the leftover via the loading and release processes can be tracked and detected.

## Conclusions

We have reported an approach of the synthesis of functional SiO_2_ · Re_2_O_3_ HSs using silica spheres, rare-earth ions, and an acidic environment. The size of synthesized hollow capsules can be modulated by controlling the diameter of the silica template. The facile and economical synthesis protocol is valuable and convenient for wide use. Acting as drug-loaded capsules, the SiO_2_ · Re_2_O_3_ HSs demonstrated much excellent properties of high payloads, retained drug activity and stability, and slow drug release rate. Furthermore, real-time detection may be carried out during drug delivery and release with SiO_2_ · Re_2_O_3_ HSs by measuring their fluorescence.

## Competing interests

The authors declare that they have no competing interests.

## Authors’ contributions

ZL is the director of the experiment group, who propounded the ideas and drafted the manuscript. LZ carried out the series of experiments and characterized all the samples. QL participated in the related experiments. YD participated in the experiment of drug loading and release. FW participated in its design and coordination. All authors read and approved the final manuscript.

## Supplementary Material

Additional file 1 **Supporting information. Table S1.** Experimental results at different reaction conditions. **Table S2.** Different Re^3+^ ion (Re = Y, Eu, La, Sm, Tb, Pr) influence on the product during synthesis process. **Figure S1.** TEM images of different reaction temperatures, [Eu^3+^] = 0.06 mol/L, 12 h. **Figure S2.** TEM images of different Eu^3+^ concentrations, 250°C, 12 h. **Figure S3.** TEM images of different pH values of solutions, 250°C, [Eu^3+^] = 0.06 mol/L, 12 h. **Figure S4.** TEM images of SiO_2_ · Re_2_O_3_ HSs prepared by different Re ^3+^ assistance: *T* = 250°C, pH = 4, [Re^3+^] = 0.06 mol/L, *t* = 12 h (Re = Y, Eu, La, Sm, Tb, Pr). Click here for file
