# Supplementary material for: A facile approach to synthesize SiO2 · Re2O3 (Re = Y, Eu, La, Sm, Tb, Pr) hollow sphere and its application in drug release
Source: Nanoscale Res Lett. 2013 Oct 21;8(1):435. doi: 10.1186/1556-276X-8-435 (PMC3819671; doi:10.1186/1556-276X-8-435)
Supplement: Additional file 1 — Supporting information. Table S1. Experimental results at different reaction conditions. Table S2. Different Re3+ ion (Re = Y, Eu, La, Sm, Tb, Pr) influence on the product during synthesis process. Figure S1. TEM images of different reaction temperatures, [Eu3+] = 0.06 mol/L, 12 h. Figure S2. TEM images of different Eu3+ concentrations, 250°C, 12 h. Figure S3. TEM images of different pH values of solutions, 250°C, [Eu3+] = 0.06 mol/L, 12 h. Figure S4. TEM images of SiO2 · Re2O3 HSs prepared by different Re 3+ assistance: T = 250°C, pH = 4, [Re3+] = 0.06 mol/L, t = 12 h (Re = Y, Eu, La, Sm, Tb, Pr). [file 1556-276X-8-435-S1.doc]

**Supporting Information**

**A Facile Approach to Synthesize SiO_2_∙Re_2_O_3_ (Re = Y, Eu, La, Sm, Tb, Pr)**

**Hollow Sphere and Its Application in Drug Release**

Zhihua Li*^1^, Lin Zhu^1^, Qian Liu^1^, Yu Du^1^, Feng Wang^2^

1. College of Chemistry, Chemical Engineering and Materials Science, Key Laboratory of

Molecular and Nano Probes, Shandong Normal University, Jinan, China, 250014

2. College of Rizhao Polytechnic, Rizhao, China, 276826

Corresponding Author: Dr. Zhihua Li, Department of Chemistry, Shandong Normal University.

E-mail: [lizhihua2006@126.com](mailto:lizhihua2006@126.com); Cellphone: +8615169118896

Table 1. Experimental results at different reaction conditions

| Sample | T (ºC) | [Eu^3+^] (mol/L) | t (hour) | pH | Results |
| --- | --- | --- | --- | --- | --- |
| Silica spheres , 0.06 g,  200 nm  (S1) | 230 | 0.06 | 12 | 4.5 | Rattle-type shperes and solid silica spheres |
|  | 240 | 0.06 | 12 | 4.5 | HSSs and a small quantity of solid silica spheres |
|  | 250 | 0.06 | 12 | 4.5 | Good quality of HSSs |
|  | 260 | 0.06 | 12 | 4.5 | HSSs, some of HSSs have been cracking |
|  | | | | | |
| Silica spheres , 0.06 g,  200 nm  (S2) | 250 | 0.05 | 12 | 4.5 | Poor quality of HSSs |
|  | 250 | 0.06 | 12 | 4.5 | Good quality of HSSs |
|  | 250 | 0.07 | 12 | 4.5 | Poor quality of HSSs and bits of rattle-type spheres |
|  | 250 | 0.08 | 12 | 4.5 | HSSs, rattle-type spheres and solid silica spheres |
|  | | | | | |
| Silica spheres , 0.06 g,  200 nm | 250 | 0.06 | 6 | 4.5 | Rattle-type spheres and solid silica spheres |
|  | 250 | 0.06 | 8 | 4.5 | HSSs companied with tiny particles |
|  | 250 | 0.06 | 10 | 4.5 | HSSs companied with tiny particles and debris |
|  | 250 | 0.06 | 12 | 4.5 | Good quality of HSSs |
|  | 250 | 0.06 | 14 | 4.5 | Poor quality of HSSs |
|  | | | | | |
| Silica spheres , 0.06 g,  200 nm  (S3) | 250 | 0.06 | 12 | 3 | Solid silica spheres |
|  | 250 | 0.06 | 12 | 4 | HSSs and solid silica spheres |
|  | 250 | 0.06 | 12 | 4.5 | Good quality of HSSs |
|  | 250 | 0.06 | 12 | 5 | HSSs |
|  | 250 | 0.06 | 12 | 5.5 | HSSs |
|  | 250 | 0.06 | 12 | 6 | HSSs and solid silica spheres |


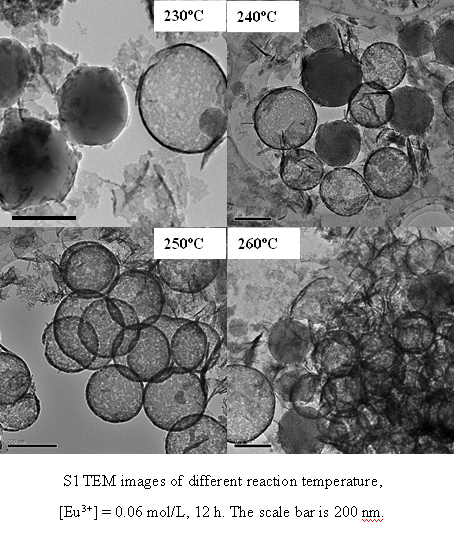


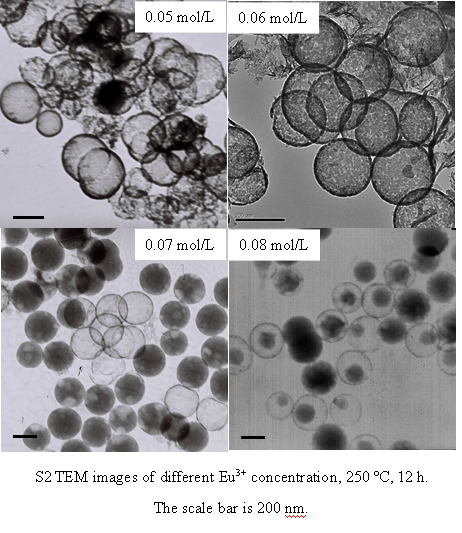


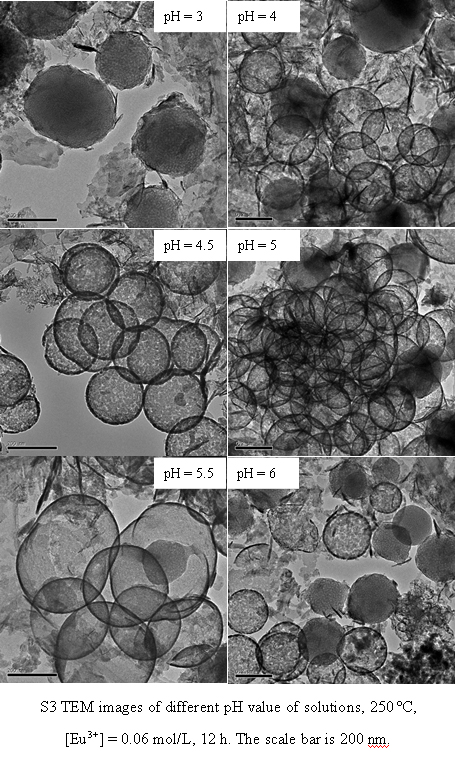


Table 2 Different Re^3+^ ions (Re = Y, Eu, La, Sm, Tb, Pr)

Influence on the product during synthesis process

| Sample | T (ºC) | pH | t (hour) | [Re^3+^]  (0.06 mol/L) | Results |
| --- | --- | --- | --- | --- | --- |
| Silica spheres , 0.06 g,  200 nm  (S4) | 250 | 4.5 | 12 | Y^3+^ | HSSs and solid silica spheres |
|  | 250 | 4.5 | 12 | Eu^3+^ | HSSs with good quality |
|  | 250 | 4.5 | 12 | La^3+^ | HSSs, solid silica spheres and debris |
|  | 250 | 4.5 | 12 | Sm^3+^ | HSSs |
|  | 250 | 4.5 | 12 | Tb^3+^ | A few HSSs, rattle-type spheres and a lot of solid silica spheres |
|  | 250 | 4.5 | 12 | Pr^3+^ | HSSs, a few solid silica spheres, tiny particles and debris |


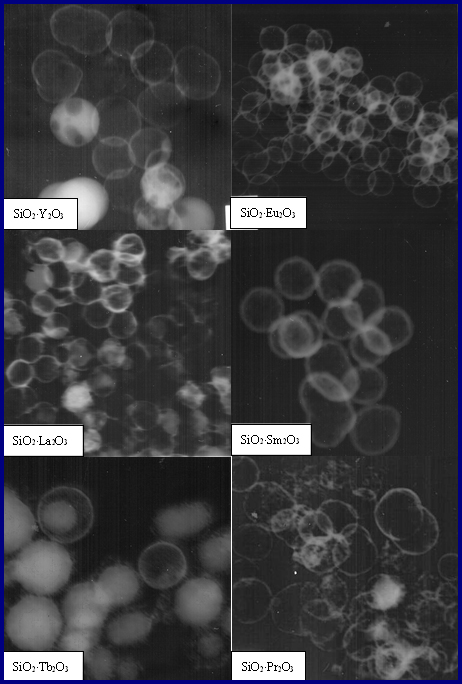


S4 TEM images of SiO_2_∙Re_2_O_3_ HSs prepared by different Re^3+^ assistance; T= 250ºC, pH = 4，[Re^3+^]=0.06 mol/L, t =12 h

(Re = Y, Eu, La, Sm, Tb, Pr)
